# Overexpression of *Prunus DAM6* inhibits growth, represses bud break competency of dormant buds and delays bud outgrowth in apple plants

**DOI:** 10.1371/journal.pone.0214788

**Published:** 2019-04-09

**Authors:** Hisayo Yamane, Masato Wada, Chikako Honda, Takakazu Matsuura, Yoko Ikeda, Takashi Hirayama, Yutaro Osako, Mei Gao-Takai, Mikiko Kojima, Hitoshi Sakakibara, Ryutaro Tao

**Affiliations:** 1 Laboratory of Pomology, Graduate School of Agriculture, Kyoto University, Kyoto, Japan; 2 Division of Apple Research, Institute of Fruit Tree and Tea Science, NARO, Morioka, Japan; 3 Institute of Plant Science and Resources, Okayama University, Kurashiki, Japan; 4 Agricultural Experimental Station, Ishikawa Prefectural University, Nonoichi, Japan; 5 RIKEN Center for Sustainable Resource Science, Tsurumi, Yokohama, Japan; Purdue University, UNITED STATES

## Abstract

Most deciduous fruit trees cultivated in the temperate zone require a genotype-dependent amounts of chilling exposure for dormancy release and bud break. In Japanese apricot (*Prunus mume*), *DORMANCY-ASSOCIATED MADS-box 6* (*PmDAM6*) may influence chilling-mediated dormancy release and bud break. In this study, we attempted to elucidate the biological functions of *PmDAM6* related to dormancy regulation by analyzing *PmDAM6*-overexpressing transgenic apple (*Malus* spp.). We generated *35S*:*PmDAM6* lines and chemically inducible overexpression lines, *35S*:*PmDAM6-GR*. In both overexpression lines, shoot growth was inhibited and early bud set was observed. In addition, *PmDAM6* expression repressed bud break competency during dormancy and delayed bud break. Moreover, *PmDAM6* expression increased abscisic acid levels and decreased cytokinins contents during the late dormancy and bud break stages in both *35S*:*PmDAM6* and *35S*:*PmDAM6-GR*. Our analysis also suggested that abscisic acid levels increased during dormancy but subsequently decreased during dormancy release whereas cytokinins contents increased during the bud break stage in dormant Japanese apricot buds. We previously revealed that *PmDAM6* expression is continuously down-regulated during dormancy release toward bud break in Japanese apricot. The *PmDAM6* expression pattern was concurrent with a decrease and increase in the abscisic acid and cytokinins contents, respectively, in dormant Japanese apricot buds. Therefore, we hypothesize that *PmDAM6* represses the bud break competency during dormancy and bud break stages in Japanese apricot by modulating abscisic acid and cytokinins accumulation in dormant buds.

## Introduction

Perennial woody plants in temperate zones synchronize their annual growth patterns with seasonal environmental changes. This allows plants to avoid injuries from environmental stresses, such as cold conditions in the winter. Dormancy is a control mechanism that enables woody perennials to adapt to seasonal environmental changes. Bud dormancy refers to the inability of the meristem to resume growth [1]. Lang [2] and Lang et al. [3] defined plant dormancy as “the temporary suspension of visible growth of any plant structure containing a meristem” and classified the fruit tree bud dormancy states as paradormancy, endodormancy, and ecodormancy. Both paradormancy and endodormancy are states induced by the perception of environmental or endogenous signaling cues, but they differ regarding whether they originated solely from meristem-containing tissue (endodormant) or from a structure distinct from the one undergoing dormancy (paradormant). A certain amount of chilling exposure is critical for inducing the shift from endodormancy to ecodormancy. Ecodormancy is a state brought about by the limitation of growth-promoting factors, such as warm conditions and the availability of water and nutrients. Although Lang’s definition has been widely adopted by researchers of plant bud dormancy, recently accumulated data suggest this terminology may need to be revised [4, 5]. For example, discriminating between paradormancy and endodormancy, and endodormancy and ecodormancy, is problematic for fruit tree species of the genus *Prunus*, such as peach (*P*. *persica*) and Japanese apricot (*P*. *mume*), in which lateral flower and leaf buds are often used for dormancy research. In these fruit tree species, whether buds are paradormant, endodormant or ecodormant has been estimated according to the competency of bud break, which is often based on the mean time to bud break or bud break percentage in forcing condition [4, 6, 7, 8, 9]. Without the experiments such as incubating branch or single node cuttings in forcing condition, we cannot precisely distinguish paradormancy, endodormancy and ecodormancy in field trees.

The genetic and molecular regulation of bud dormancy has been extensively studied in the model woody perennial, poplar (*Populus* spp.), and much has been learned, as reviewed [1, 10, 11]. Additionally, molecular networks regulating the dormancy of various woody species, including horticulturally important fruit tree species, have been characterized based on omics studies of specific plant species [4]. In a previous study of Japanese apricot, which belongs to the family Rosaceae, Yamane et al. [12] applied an RNA subtraction technique to identify the genes expressed preferentially in endodormant buds (no bud break under forcing conditions) compared with paradormant (higher bud break frequency under forcing conditions compared with that of endodormant buds) and ecodormant buds (bud break frequency greater than 50% under forcing conditions). This study identified a MADS-box gene with dormancy-associated expression. This gene was similar to the *Arabidopsis thaliana StMADS11* clade, which includes SHORT VEGETATIVE PHASE (*SVP*) and AGAMOUS-LIKE24 (*AGL24*) [12]. In peach, a mutant that continues to grow and fails to enter dormancy under dormancy-inducing conditions has been identified. This mutant, which is known as *evergrowing* (*evg*; USDA PI442380), was first identified in southern Mexico [13]. Sequencing and expression analyses of the *evg* locus identified six *StMADS11* (*SVP/AGL24*)-clade MADS-box genes that may be associated with terminal bud formation [14]. These genes were named as *DORMANCY-ASSOCIATED MADS-box 1*–*6* (*DAM1*–*6*) genes. The gene that Yamane et al. [12] detected in Japanese apricot RNA subtraction study appears to be an ortholog of peach *DAM6*, and was named *PmDAM6*.

In the Japanese apricot genome, six tandemly arrayed *PmDAM* genes (*PmDAM1*–*6*) have been identified [15, 16]. A seasonal expression analysis of the *PmDAM* genes in a reverse transcription-quantitative PCR (RT-qPCR) [15], genome-wide transcriptomic analyses involving the Japanese apricot expressed sequence tag (EST) dormant bud database [17], and a 60 K-microarray analysis [18] revealed that *PmDAM* genes are preferentially expressed in dormant leaf buds, in summer (*PmDAM1-3*) and autumn (*PmDAM4-6*). Moreover, the expression levels of these genes are down-regulated during the dormancy release toward bud break of the lateral leaf buds. Furthermore, RT-qPCR and microarray analyses indicated that the expression levels of all six *PmDAM* genes are down-regulated following a prolonged exposure to artificial cold conditions [15]. Kitamura et al. [19], Zhang et al. [20], and Zhong et al. [21] also demonstrated that *PmDAM*s are expressed in endodormant flower buds in November and their expression levels are down-regulated during dormancy release and bud break. Meanwhile, Zhao et al. [22] examined flower buds, and highlighted the functions of *PmDAM*s in flower development as well as dormancy induction. The peach and Japanese apricot *DAM6* expression levels in buds are negatively correlated with increasing bud break competency (bud-burst frequency in forcing conditions). In peach, *DAM6* expression was negatively correlated with the time required for terminal bud break [23]. A negative correlation between peach *PpDAM6* expression and the time required for bud break under forcing conditions was also reported for lateral leaf [24] and flower [25, 26] buds. Among the other rosaceous perennials and temperate fruit trees, the down-regulated expression of the *DAM*-like genes during dormancy release and bud break has been reported for the lateral buds of raspberry (*Rubus idaeus* L.) [27], the lateral leaf and flower buds of pear (*Pyrus pyrifolia*) [28, 29], and the terminal buds of apple (*Malus* × *domestica*). In apple, Porto et al. [30] proposed that the ‘Golden Delicious’ apple genome contains four *DAM*-like genes (*MdDAM1–4*). Expression analyses suggested that the *MdDAM* genes undergo seasonal expression-level changes, with an up-regulation during endodormancy [30–33]. The overexpression of *MdDAMb*, which is a *SVP/DAM*-like gene and up-regulated during ecodormancy [34], delays bud break [35], suggesting that *DAM* genes may affect bud break in both apple and *Prunus* spp.

Most studies regarding *Prunus DAM* genes have focused on expression levels, and have revealed a correlation between *DAM* expression levels and dormancy depth (days to bud break under forcing conditions). Among the *DAM*s in Japanese apricot, *PmDAM6* is the most promising candidate regulator of dormancy because its expression is correlated with the endodormancy release and ecodormancy release (increased bud break frequency under forcing condition). Moreover, the results of our earlier comprehensive transcriptome analysis suggested that *PmDAM6* showed the biggest difference in the expression-level fold change by artificial chilling exposure [17, 18]. Thus, validating the function and clarifying the downstream factors regulated by *PmDAM6* are necessary to characterize the mechanism controlling dormancy in Japanese apricot buds. To elucidate the biological functions of *PmDAM6*, hybrid poplar (*Populus tremula* × *Populus tremuloides*; clone T89) plants constitutively expressing *PmDAM6* under the control of the cauliflower mosaic virus 35S promoter (*35S*:*PmDAM6*) were generated. Additionally, their phenotypes were compared with those of control plants, which were either wild-type poplar or poplar transformed with the empty vector [15]. When grown under long photoperiod (LP) conditions (16-h light/8-h dark), the shoot growth of *35S*:*PmDAM6* poplars was inhibited. Additionally, *35S*:*PmDAM6* poplars set terminal buds earlier than the control poplars. Thus, *PmDAM6* may function as a growth inhibitor in poplar and induce bud set, which is a dormancy-related trait. We then attempted to obtain Japanese apricot overexpressed or RNA interference (RNAi)-suppressed transformants. However, we obtained an insufficient number of lines for a meaningful analysis of phenotypes [36]. Therefore, in this present study, we used transgenic apples to functionally validate the role of *PmDAM6* because Japanese apricot is phylogenetically closer to apple than to poplar. Moreover, easily transformed apple lines are available. Additionally, the existence of *MdDAM*s, which are phylogenetically similar to *Prunus DAM*s, imply that similar *DAM*-controlled downstream factors and pathways might be conserved in apple and *Prunus* species. Thus, apple transformants may be useful for a detailed analysis of the biological functions of *Prunus* DAMs. On the basis of the observed phenotypes and hormone contents in the dormant buds of transformed *PmDAM6*-overexpressing apple lines, we herein discuss the biological functions of *PmDAM6* and present a working hypothesis regarding how *PmDAM6* regulates dormancy and bud break in Japanese apricot.

## Materials and methods

### Apple transformation

In this study, we used the binary vectors *p35S*:*PmDAM6* [15] and *p35S*:*PmDAM6-GR*, in which the *PmDAM6* of the former vector was substituted by a fusion between PmDAM6 and the hormone-binding domain of a mouse glucocorticoid receptor [37]. In *p35S*:*PmDAM6-GR* transgenic apples, PmDAM6-GR was localized in the cytoplasm in the absence of glucocorticoid, and was recruited to the nuclei only when cells were treated with dexamethasone (DEX), which is a glucocorticoid compound. These vectors were introduced into *Agrobacterium tumefaciens* strain LBA4404, which were subsequently used to transform an apple rootstock cultivar, ‘JM2’ [38]. Apple transformations were conducted as described by Wada et al. [39]. *Agrobacterium tumefaciens* cells was cultured overnight on a shaker in 20 mL of ψB medium with 50 mg/L kanamycin at 28°C. After a centrifugation, the pellet was resuspended in half-strength Murashige and Skoog (MS) medium, and further diluted to an optical density at 600 nm (OD_600_) of 0.8. Folded small leaf explants were soaked in the *Agrobacterium* solution, immediately sonicated (UT-105S; Sharp Corporation, Osaka, Japan) for 10 s, and incubated for 15 min. Then, the inoculated explants were incubated for 1 week on the co-cultivation medium N6+MS basic medium (pH 5.6) [38] containing 0.2 mg L^−1^ NAA, 5 mg L^−1^ BAP, 30 g L^−1^ sorbitol, 2.6 g L^−1^ phytagel (Sigma-Aldrich, St. Louis, MO, USA), and 20 μM acetosyringone, at 22°C in the dark. The explants were transferred to a selective medium, which was the same as the co-cultivation medium except it contained 25 mg L^−1^ kanamycin and 50 mg L^−1^ meropenem trihydrate (MEROPEN; Dainippon Sumitomo Pharma Co., Ltd., Osaka, Japan) and lacked acetosyringone. After 1 month, explants were incubated at 22°C under a 16-h light/8-h dark photoperiod and were subcultured monthly. Regenerated shoots were grown on proliferation medium [basic MS medium (pH 5.8) containing 0.1 mg L^−1^ indole-3-butyric acid, 0.5–1 mg L^−1^ 6-benzylaminopurine, 30 g L^−1^ sucrose, 7 g L^−1^ bactoagar, 25 mg L^−1^ kanamycin, and 50 mg L^−1^ meropenem trihydrate]. The regenerated shoots were transferred to sterilized vermiculite moistened with the basic MS medium supplemented with 0.1 mg L^−1^ indole-3-butyric acid and 30 g L^−1^ sucrose (pH 5.8). When the plants rooted, they were moved to plastic pots containing sterilized vermiculite moistened with 1/1,000 Hyponex (Hyponex Japan, Osaka, Japan) and covered with a plastic bag. After several weeks of acclimatization, the plastic bags were removed, and the plants were transplanted to larger pots (10.5 cm diameter Y pot, Sakata Seed Corporation, Yokohama, Japan). All plants were cultivated in a growth chamber or greenhouse with standard procedures, including pesticide, fungicide sprays, and fertilizer treatments.

The success of apple transformations was confirmed by a DNA blot analysis as previously described [40]. Briefly, genomic DNA was isolated from leaves with a plant DNA isolation kit (NR-501; Kurabo, Osaka, Japan). Genomic DNA was digested with *Hin*dIII, run on a 0.8% agarose gel, and transferred to a Biodyne PLUS membrane (Pall, Port Washington, NY, USA). The membrane was hybridized with a digoxigenin-labeled *PmDAM6* probe [36].

After an acclimatization step, apple plants from two transgenic lines, *35S*:*PmDAM6* (35S-2 and 35S-4) and *35S*:*PmDAM6-GR* (GR21 and GR22), as well as wild-type (WT) control apple plants (n = 10–17 per line) were grown under LP conditions (22°C with a 16-h light/8-h dark photoperiod) for 4 months beginning in December 2011. In May 2012, after all plants had stopped growing, leaves were collected for a subsequent analysis of *PmDAM6* expression levels. Total RNA was isolated, and 1 μg was used as the template for a cDNA synthesis step, as described previously [15]. The cDNA solution synthesized from approximately 20 ng of total RNA was used for RT-qPCR, which was performed using LightCycler 480 (Roche, Basel, Switzerland) and a probe master mix (Roche). Primers and probes used for the amplification of *PmDAM6* [15] are shown in S1 Fig. As a reference, an apple *SAND* (*MDP0000185470* and/or *MDP0000202305*) [41] gene was monitored by RT-qPCR using SYBR Green Master mix (Roche) and gene-specific primers for *MdSAND* (S1 Table) for normalization. PCR was performed using a program of 45 cycles at 94°C for 10 s, 55°C for 20 s, and 72°C for 1 s, with initial denaturing at 95°C for 5 min. For *SAND* gene-specific qPCR, a dissociation curve analysis was performed to confirm that the fluorescence was only derived from gene-specific amplification. Three biological replicates were analyzed.

### Observation of growth and bud dormancy states of *35S*:*PmDAM6* apple plants

In May 2012, all 2-year-old transgenic plants were moved to a closed greenhouse in Kyoto, Japan and cultivated under a natural photoperiod. The greenhouse was cooled when the temperature exceeded 25°C (May to September) or 15°C (October to April). The greenhouse was not heated throughout the experimental period. From April to July 2013, three 3-year-old plants from each line were moved from a closed greenhouse to growth chamber and incubated under short photoperiod (SP) conditions (20°C with an 8-h light/16-h dark photoperiod) for 3 months. Plant height and the timing of bud set were monitored. The remaining plants were grown in a greenhouse.

For each line grown in the greenhouse with naturally occurring cold conditions, five plants were labeled as standard plants, and their terminal bud set timing, leaf shedding timing, and bud break dates were monitored annually from the 2013–14 season (3-year-old) through to the 2016–17 season (6-year-old). The number of lateral buds that had burst was determined in the 2013–14 (3-year-old) and 2014–15 seasons (4-year-old), but there were too many lateral buds on the lateral shoots in the 2015–16 season (5-year-old) onward to be accurately counted. Plants were photographed on 1 April 2014 (4-year-old), 24 July 2014 (4-year-old), 28 August 2015 (5-year-old) and 15 April 2016 (6-year-old).

In December 2015, 5-year-old WT and 35S-4 (n = 3) plants grown in the greenhouse were moved to a cold room (5 °C in darkness). After a 30-day or 60-day cold treatment, the plants were exposed to forcing conditions (> 15 °C in a greenhouse). The number of days to bud break was determined for the terminal buds. This experiment was repeated in December 2016 using 6-year-old plants.

On 23 February and 8 March, 2017, the current year’s shoots that were approximately 15 cm long (n = 5; 1 to 2 shoots each from 3 plants per genotype) were collected from 6-year-old 35S-4 and WT plants. The basal parts of shoots were soaked in water containing 1% Misakifarm (OAT Agrio, Tokyo, Japan), which is a cut-flower freshness preservation agent containing nutrients and fungicides, and then incubated under forcing conditions (22 °C with a 16-h light/8-h dark photoperiod). The basal parts of shoots were cut once per week to promote water uptake, and the solution was replaced weekly. The bud break rate (%) was recorded for 1 month.

For the phytohormone analysis, terminal buds were collected from greenhouse-grown 4-year-old plants on 20 February 2015, 6-year-old plants on 25 January 2017, 23 February 2017, 8 March 2017 and 31 March 2017, after which they were immediately frozen in liquid N_2_ and stored at −80 °C until analyzed.

### Observation of bud dormancy status of DEX-treated *35S*:*PmDAM6-GR* apple plants

In November 2013, after the trees had shed their leaves, 3-year-old GR21, GR22, and WT plants (n = 3–5) were pruned, leaving only one main shoot from the current year, and moved from the greenhouse to a cold room (5°C in darkness). After 4 weeks, the plants were transferred to forcing conditions (22°C with a 16-h light/8-h dark photoperiod) and grown for 4 months. The 20 μM DEX solution containing 0.1% Tween 20 and 0.1% ethanol or the control solution containing 0.1% Tween 20 and 0.1% ethanol were applied every 3 days to the soil of pot-grown plants being grown under forcing condition. The number of days to bud break for the terminal buds and the number of lateral buds that had burst were determined. The length of the shoots sprouting from terminal buds and bud set rate (%) were recorded after 4 months under forcing conditions. Plants were photographed on 7 March 2014.

After the 2013–14 season, uniform DEX applications were not possible owing to the increasing plant sizes. Therefore, we allowed the branches to take up the DEX solution by soaking the basal parts of branches in 20 μM DEX. This treatment method prevented us from observing phenotypes over long periods. Dormant shoots require relatively long incubation periods for the to uptake liquids taken up at the basal parts of cut branches to reach the terminal buds and influence phenotypes. Thus, we were unable to determine the effects of DEX on the dormancy stage. Consequently, we tested the effects of DEX on dormant buds during the bud break stage. On 6 March 2017, the current year’s shoots that were approximately 15 cm long (n = 6–10, 1 to 2 shoots from 5 plants per genotype) were collected from 6-year-old GR21, GR22, and WT plants. The basal parts of shoots were soaked in 20 μM DEX or control solutions and incubated under forcing conditions (22°C with 16-h light/8-h dark photoperiod). For the phytohormone analysis, terminal buds were collected after 0, 24, and 96 h for GR21 and after 0 and 96 h for WT under forcing conditions, immediately frozen in liquid N_2_, and stored at –80 °C until analyzed. After a 7-day incubation, the bottom parts of shoots were cut to promote water uptake, and solution was replaced by a fresh solution. After a 15-day incubation, the shoots sprouting from buds were photographed, and the blade lengths of the three most developed leaves sprouting from the buds were measured.

### Investigation of the phytohormone contents of the terminal buds of transgenic apple plants

Terminal bud samples were freeze-dried and ground to a fine powder using a Multi-beads shocker (Yasui Kikai Co., Osaka, Japan). For *35S*:*PmDAM6* apple plants, plant hormones were extracted from 20-mg samples in a methanol–formic acid buffer (methanol:water:formic acid = 15:4:1) containing a deuterium-labeled hormone internal standard [i.e., 40 ng d_6_-abscisic acid (ABA); 4 ng d_5_-indole acetic acid (IAA); 0.4 ng d_5_-trans zeatin (tZ), and d_6_-isopentenyl adenine (iP)]. Hormones were extracted overnight at 4 °C in darkness. Supernatants were purified with Oasis HLB columns (Waters Co., Milford, MA, USA) and then evaporated. The dry resulting pellets were resuspended in 1 M formic acid and then added to Oasis MCX columns (Waters). Acid and basic fractions were extracted in 100% methanol and 0.35% NH_3_ in 60% methanol, respectively. Samples were evaporated, and the remaining pellets were resuspended in reconstitution solution (acetonitrile:water:formic acid = 85:15:0.1). Phytohormone contents were determined by liquid chromatography–triple quadrupole mass spectrometry (Waters; liquid chromatography system: Waters 2695, mass spectrometer: Quattro micro ARI Waters 2996).

For GR21 apple plants, phytohormone contents were analyzed as previously described [42] with minor changes. Briefly, harvested tissue samples were lyophilized and the exact dry weights (around 20 mg per sample) were determined. To avoid the effects of the ion suppression of salicylic acid (SA) on apple bud samples, we modified the solution used during the SA elution step. After sample purification by HLB and MCX columns, the acidic fraction was loaded onto an Oasis WAX column (Waters), and the main acidic fraction was eluted with 1% AcOH and 80% MeCN. A solution comprising 3% formic acid and 97% MeCN was then applied to the WAX column to elute SA. The eluates were evaporated to dryness, reconstituted in 1% AcOH and subjected to phytohormone quantification using an Agilent 1260–6410 Triple Quad LC/MS system (Agilent Technologies Inc., Santa Clara, CA, USA) equipped with a ZORBAX Eclipse XDB-C18 column and an XDB-C8 Guard column (Agilent Technologies Inc.). In this analysis, IAA, tZ, iP, ABA, jasmonic acid (JA), JA-isoleucine (JA-Ile), and SA contents were determined.

### Investigation of phytohormone contents in dormant leaf and flower buds of Japanese apricot

Japanese apricot ‘Nanko’ grown at the experimental farm of Kyoto University, Kyoto, Japan (34°N, 135°E) were used in this study. Leaf buds and flower buds collected monthly from September to March during the 2005–06 were immediately frozen in liquid nitrogen and stored at −80°C until used for plant hormone analyses. Phytohormone contents were determined as previously described [43] without MS Probe modification. In this analysis, IAA, tZ, trans zeatin riboside (tZR), iP, isopentenyl adenine riboside (iPR), and ABA contents were determined.

## Results

### Shoot growth and dormancy induction phenotypes of *35S*:*PmDAM6* and *35S*:*PmDAM6-GR* apple plants

When the *p35S*:*PmDAM6* binary vector was used to transform the apple rootstock cultivar ‘JM2’, the transformation efficiency was 0.25%, which was approximately 10 times lower than usual (approximately 1%–5%). We obtained two *35S*:*PmDAM6* lines, 35S-2 and 35S-4. A genomic DNA blot analysis using the *PmDAM6* probe indicated that a single copy was inserted into each transgenic line (S1 Fig). Additionally, *PmDAM6* mRNA was detected in both 35S-2 and 35S-4, with a higher expression level in 35S-4 than in 35S-2 (S1 Fig). Moreover, *PmDAM6-GR* mRNA was also detected in two *35S*:*PmDAM6-GR* lines used in this study.

**Fig 1:**
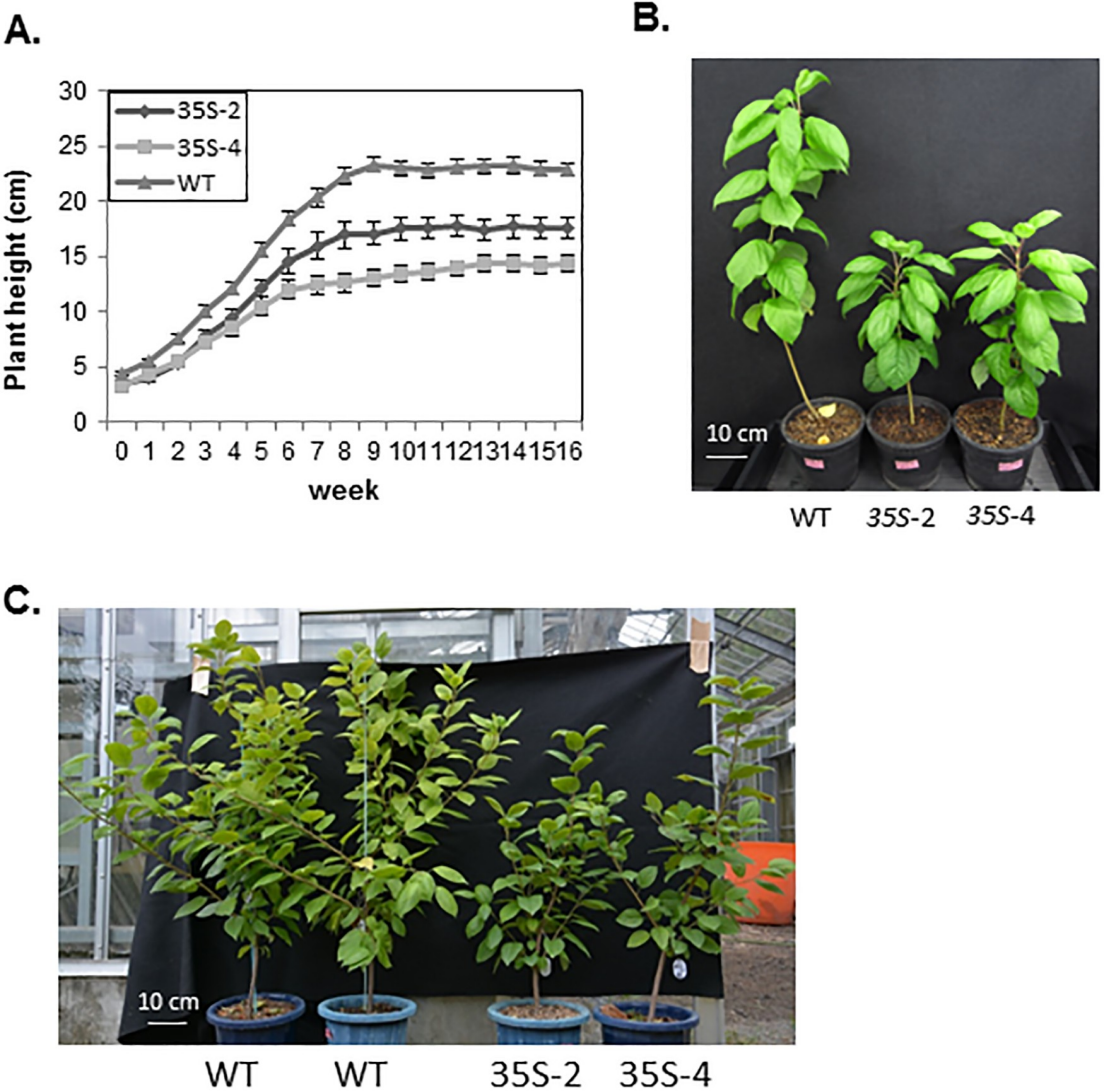
Inhibited shoot growth and reduced plant size of *35S*:*PmDAM6* apple plants. (A) Shoot growth was inhibited in the 2-year-old *35S*:*PmDAM6* lines, 35S-2 and 35S-4, relative to the growth of wild-type (WT) plants, under long photoperiod (16-h light/8-h dark) conditions. Plant height of 35S-2 and 35S-4 were significantly lower (P < 0.01) than those of WT in all examined periods (Student’s *t*-test) (B) Photographs of 3-year-old plants taken after they stopped growing under short photoperiod (SP) (8-h light/16-h dark) conditions. *35S*:*PmDAM6* plants were shorter than the WT plants (right). (C) Photograph of 5-year-old plants taken on 28 August 2015. The *35S*:*PmDAM6* plants were shortened than the WT plants. Plants were grown in a greenhouse without heating, resulting in exposure to naturally occurring cold conditions in the winter.

Shoot growth was inhibited in 2-year-old *35S*:*PmDAM6* plants under LP conditions (Fig 1A) and in 3-year-old plants under SP conditions (Fig 1B). The shoots stopped growing in all *35S*:*PmDAM6* and WT plants terminated their shoot growth in even under LP conditions. The node numbers and internode lengths were significantly lower in the *35S*:*PmDAM6* plants than in the WT plants (P < 0.01) (S2 Table). There was no major difference between the *35S*:*PmDAM6* and WT plants regarding the exact timing of terminal bud set even under SP condition. When *35S*:*PmDAM6* and WT plants were exposed to a cool conditions (10°C), leaf shedding was observed at around the same time. Reduced plant size of *35S*:*PmDAM6* plants compared to WT plants were observed even in 5-year-old plants (Fig 1C).

**Fig 2:**
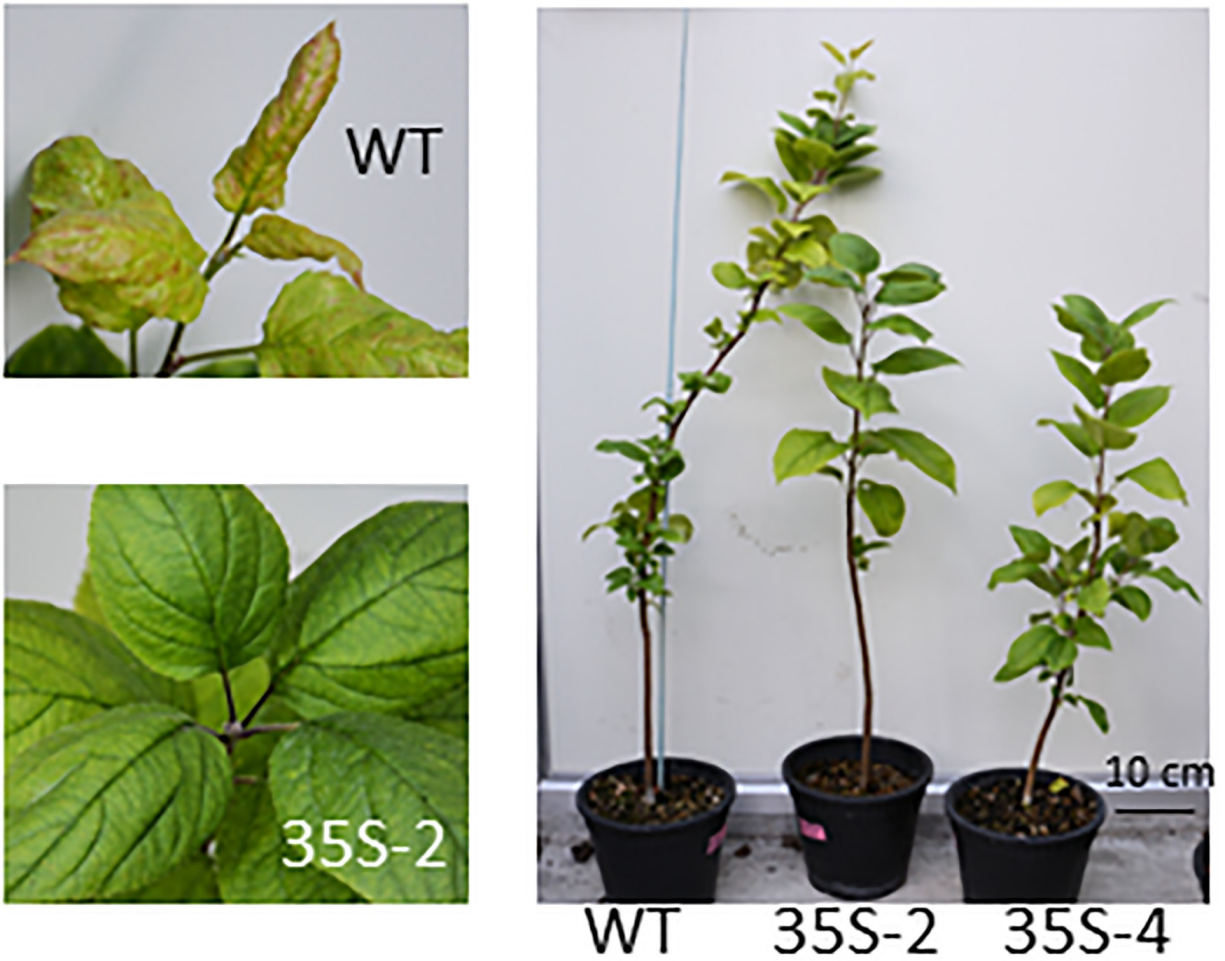
Early bud set observed in 4-year-old *35S*:*PmDAM6* apple plants. Photographs of 4-year-old plants taken on 24 July 2014. Early bud set was observed in 35S-2 plants (left).

In contrast to the 2- and 3-year-old plants, 4-year-old *35S*:*PmDAM6* plants set terminal buds approximately 1 week earlier than WT plants (Fig 2). Inhibited shoot growth and earlier bud set was also observed in 4-year-old *PmDAM6* activity-inducible transgenic lines, GR21 and GR22, when they were transferred to forcing conditions after bud break and treated with DEX continuously for 4 months (S3 Table). DEX treatment did not affect these traits in WT plants (S3 Table). The timing of leaf shedding for the *35S*:*PmDAM6* plants was similar to that of WT plants throughout the observation period.

### Bud break phenotypes and the dormancy depth of transgenic apple plants overexpressing *PmDAM6*

After plants had adapted to the growing conditions in a greenhouse without additional heating (semi-field conditions) in the Kyoto, Japan climate, we determined terminal bud break dates for 3- to 6-year-old *35S*:*PmDAM6* plants annually for 4 consecutive years from the 2013–14 season to the 2016–17 season.

**Fig 3:**
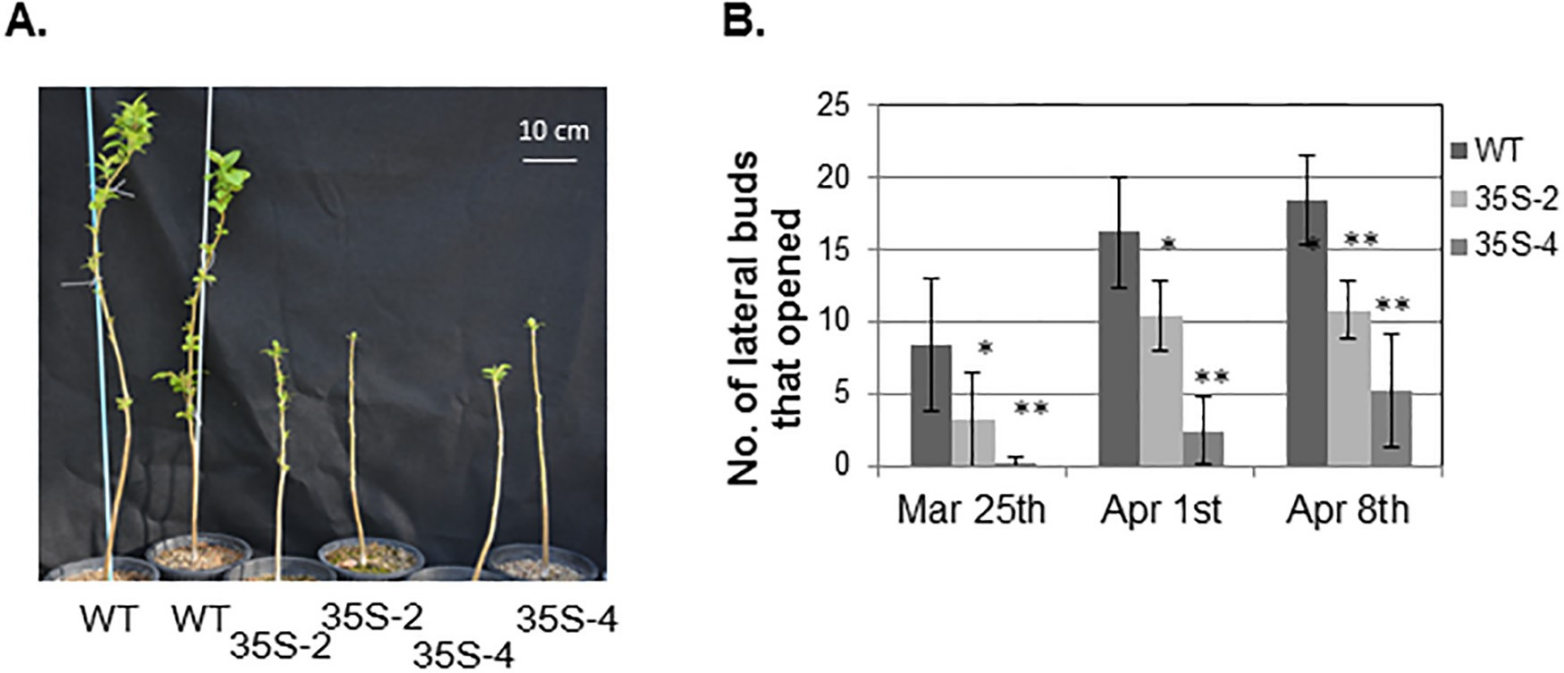
Inhibition of lateral bud break in 3-year-old *35S*:*PmDAM6* apple plants during the spring in a greenhouse without heating (semi-field conditions). (A) Photo taken on 1 April 2014. (B) Number of lateral buds that opened by 25 March, 1 April, and 8 April 2014 in the *35S*:*PmDAM6* apple lines, 35S-2 and 35S-4, and wild-type (WT) plants. Significant differences between *35S*:*PmDAM6* and WT at P < 0.01 and P < 0.05 (Student’s *t*-test) are indicated with ** and *, respectively.

For 3-year-old plants, *PmDAM6* inhibited only lateral bud break and rather accelerated terminal bud break. Terminal bud break of *35S*:*PmDAM6* plants, 35S-2 and 35S-4 occurred at 4.0 ± 5.0 and 8.0 ± 7.7 days after 28 February, respectively, whereas that of WT occurred at 16.0 ± 10.0. Earlier terminal bud break was observed in *35S*:*PmDAM6* plants compared to WT plants, but the difference was not significant (S4 Table). There were fewer open lateral buds in *35S*:*PmDAM6* plants than in WT plants (Fig 3A and 3B, S4 Table). Furthermore, for 3-year-old 35S:PmDAM6-GR plants, terminal bud break occurred significantly earlier in DEX-treated plants than in the control plants (Table 1). Additionally, significantly fewer lateral buds underwent bud break in DEX-treated plants than in control plants (Table 1).

**Table 1:** Accelerated terminal bud break and inhibition of lateral bud break in 3-year-old *35S*:*PmDAM6* and *35S*:*PmDAM6-GR* apple plants treated with DEX.

	*35S*:*PmDAM6-GR* line# 21 (GR21)		*35S*:*PmDAM6-GR* line# 22 (GR22)		WT	
	DEX	Control	DEX	Control	DEX	Control
Terminal bud break date^a^	38.1 ± 2.2 (n = 9)	49.0 ± 5.7 (n = 10)	37.6 ± 1.8 (n = 8)	46.3 ± 7.1 (n = 8)	44.7 ± 9.3 (n = 3)	41.0 ± 5.2 (n = 4)
Significant difference^b^	**		**		n.s.	
Number of opened lateral buds^c^	0 (n = 9)	3.0 ± 2.1 (n = 10)	0.1 ± 0.4 (n = 8)	2.6 ± 1.8 (n = 8)	4.3 ± 1.2 (n = 3)	3.0 ± 1.4 (n = 4)
Significant difference^b^	**		**		n.s.	

^a^Terminal bud break timing was determined by counting the days to bud break after plants chilled for 4 weeks were transferred to forcing conditions (> 15 °C in a greenhouse).

^b^Significant difference between DEX and control treatments (P < 0.01; Student’s t-test) is indicated with **, n.s., not significant.

^c^Number of sprouted lateral buds counted after 8 weeks under forcing conditions

**Fig 4:**
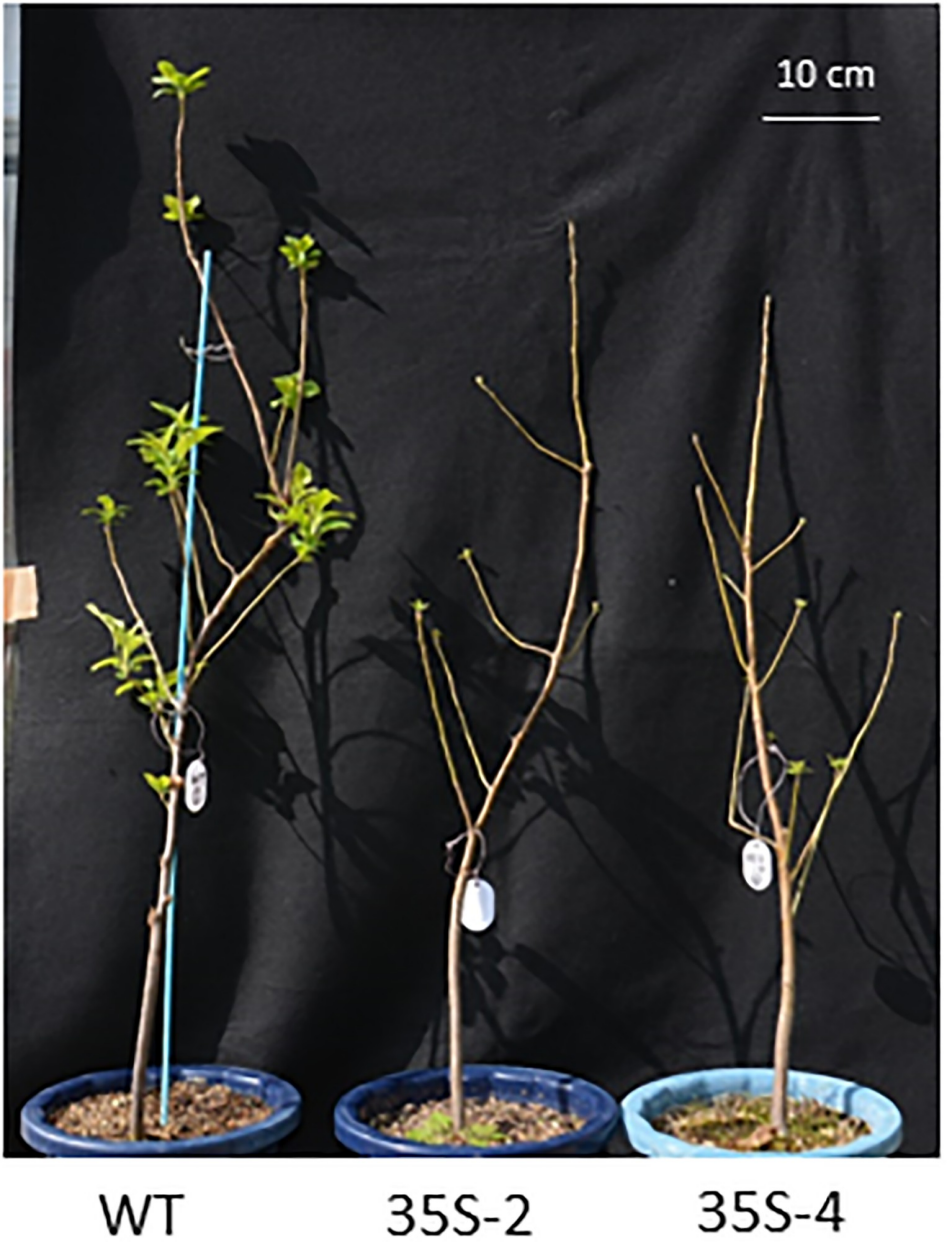
Terminal bud break in the spring was delayed in 5-year-old *35S*:*PmDAM6* apple plants. Photograph taken on 15 April 2016. Plants were grown in a greenhouse without heating, resulting in an exposure to naturally occurring cold conditions in the winter.

The bud break dates were then continuously observed in 4- to 6-year-old *35S*:*PmDAM6* plants grown under semi-field conditions from the 2014–15 season to the 2016–17 season. In contrast to 3-year-old plants, the number of open lateral buds was similar between the 4-year-old *35S*:*PmDAM6* and WT plants. Additionally, terminal bud break occurred later in the *35S*:*PmDAM6* plants than in the WT plants during these 3 consecutive years (Table 2, Fig 4).

**Table 2:** Delayed bud break timing and repressed bud break competency of dormant terminal buds of 4- to 6-year old *35S*:*PmDAM6* apple plants.

Plant age or treatment	WT	*35S*:*PmDAM6* (35S-2)^c^^,^^d^	*35S*:*PmDAM6* (35S-4)^c^
*Terminal bud break date*^a^			
four-year-old plants	2.0 ± 2.0	13.4 ± 5.0**	15.8 ± 9.7**
five-year-old plants	2.4 ± 2.2	8.8 ± 2.7**	12.4 ± 2.2**
six-year-old plants	6.8 ± 5.2	13.2 ± 5.2	18.6 ± 4.1**
*Bud break competency of dormant buds*^b^			
*Five-year-old plants*			
After thirty days of chilling treatment	28.0 ± 0.0	n.t.	48.7 ± 6.6*
After sixty days of chilling treatment	18.0 ± 0.0	n.t.	25.7 ± 2.9
*Six-year-old plants*			
After thirty days of chilling treatment	30.3 ± 3.8	n.t.	52.7 ± 7.4*
After sixty days of chilling treatment	17.3 ± 2.1	n.t.	26.0 ± 2.8*

^a^Days after 6 March 2015, 1 April 2016, and 23 March 2017, were counted for 4-, 5-, and 6-year old plants, respectively.

^b^Chilling requirements for bud break were determined by counting the days to terminal bud break after chilled plants were transferred to forcing conditions (> 15 °C in a greenhouse).

^c^Significant differences between 35S:PmDAM6 and WT plants at P < 0.01 and P < 0.05 (Student’s t-test) are indicated with ** and *, respectively.

^d^n.t., not tested

The dormancy depth of terminal buds was estimated by counting the days to bud break under forcing conditions of 35S-4 and WT plants using 5- and 6-year-old plants in the 2015–16 and 2016–17 seasons, respectively. As shown in Table 2, dormancy was significantly deeper (P < 0.01) in 35S-4 plants than in WT plants after the same amount of chilling exposure. In other words, bud break competency was significantly repressed in 35S-4 plants relative to that in WT plants. A similar difference was observed between 35S-4 and WT plants during the following growing season (Table 2).

### Phytohormone contents in the terminal buds of transgenic apple plants overexpressing *PmDAM6* from dormancy through the bud break stage

**Fig 5:**
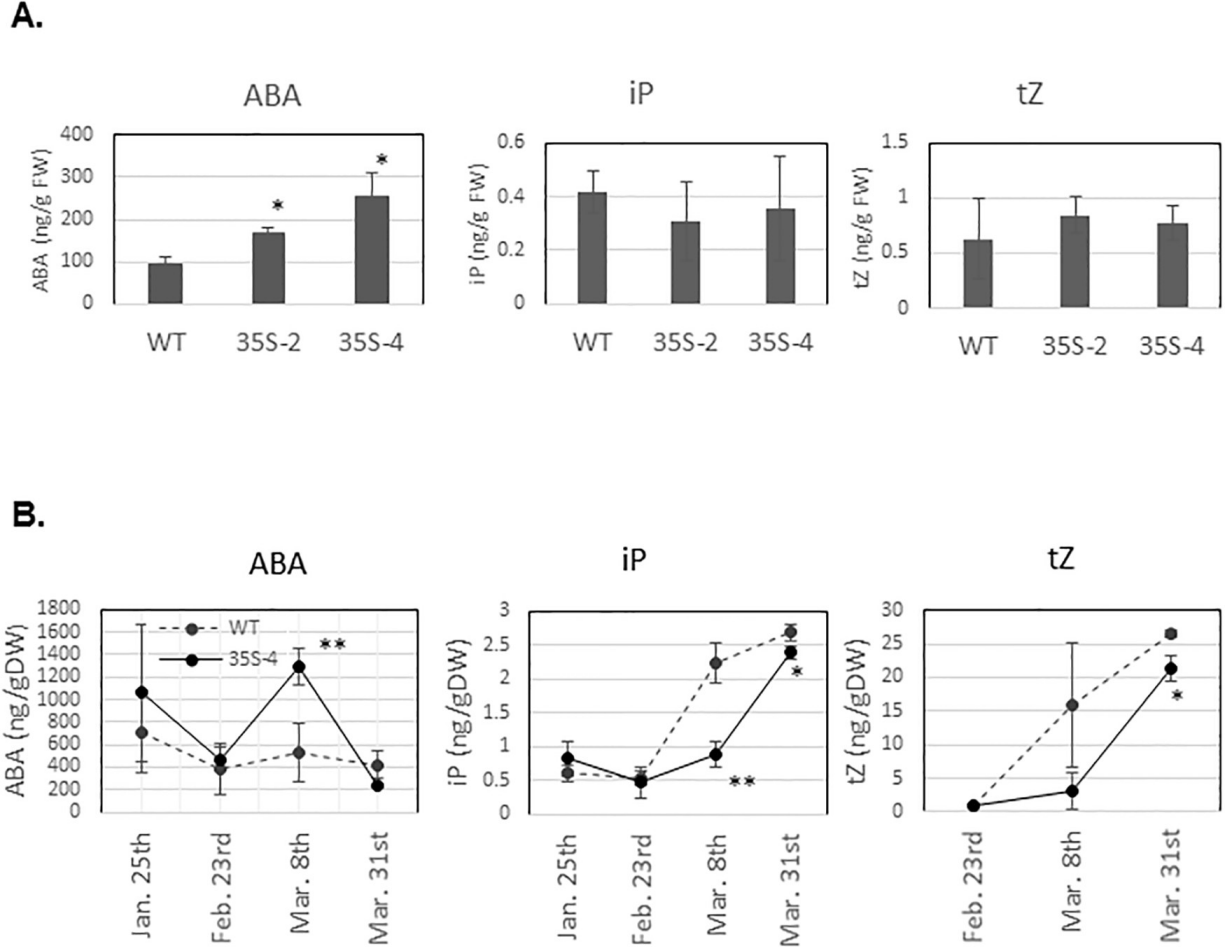
Abscisic acid and cytokinins (tZ and iP) contents in the terminal buds were higher and lower, respectively, in the dormancy-enhanced *35S*:*PmDAM6* apple plants than in the WT plants. Phytohormone contents in the terminal buds of 4-year-old plants collected on 20 February 2015 (A), and of 6-year-old plants collected on 25 January, 23 February, and 8 and 31 March 2017 (B). Values are presented as the means of three replicates. Bars indicate standard errors. Significant differences in the phytohormone contents between WT and *35S*:*PmDAM6* apple plants are indicated with ** and * at *P* < 0.01 and *P* < 0.05 (Student’s *t*-test), respectively.

The terminal buds of 4-year-old 35S-4 plants collected in February 2015 (late dormancy period) contained more ABA than the corresponding WT buds, whereas there were no significant differences in the cytokinins (CK) contents (Fig 5A). Among 6-year-old plants, the ABA content of terminal buds was greater in 35S-4 plants than in WT plants during the late dormancy stage, especially at the beginning of March (Fig 5B). In contrast, the contents of two CKs, tZ and iP, were lower in 35S-4 plants than in WT plants in March (Fig 5B). Regarding the samples collected on 25 January 2014, tZ was undetectable in both 35S-4 and WT plants. The IAA level was lower in 35S-4 plants than in WT plants, although the difference was not significant (S7 Table). The dormancy states of these buds were briefly compared by determining the days to bud break under forcing conditions. More than 50% of WT and 35–4 shoots collected on 8 March underwent bud break in 1 and 2 weeks, respectively, suggesting that the dormancy was deeper and bud break competency was lower for the 35S-4 plants than for the WT plants in March.

**Fig 6:**
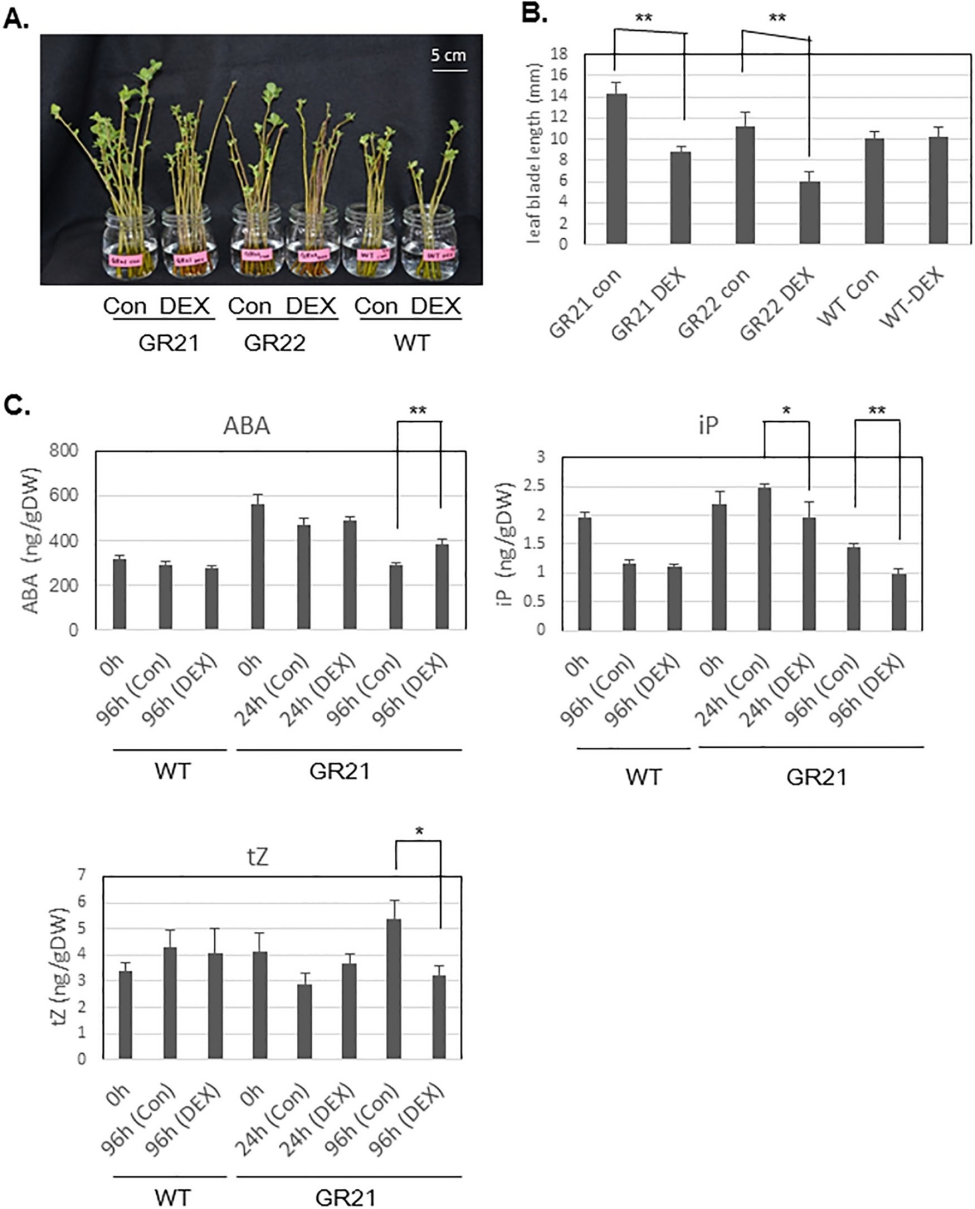
Dexamethasone (DEX) treatments inhibited bud outgrowth, increased the ABA level, and decreased cytokinin (tZ and iP) levels in the terminal buds of *35S*:*PmDAM6-GR* plants. The branches collected from 6-year-old *35S*:*PmDAM6-GR* lines, GR21 and GR22, and WT plants on 9 March, 2017 were incubated in DEX- or control solutions under forcing conditions (22°C with a 16-h light/8-h dark photoperiod). The subsequent bud outgrowth and changes in phytohormone contents were analyzed. (A) Branches were photographed at 15 days after initiating the treatment. Control (con) or DEX-treated (DEX) GR21, GR22, and WT plants are presented. (B) Average leaf blade length of the largest leaves on shoots that germinated from buds collected at 15 days after the treatment. (C) Phytohormone contents in the dormant terminal buds of branches at 0, 24, and 96 h after the treatment. Values are presented as the mean of three replicates. Bars indicate standard errors. Significant differences in the phytohormone contents of control and DEX-treated samples at the same time point in each line are indicated with ** and * at *P* < 0.01 and *P* < 0.05 (Student’s *t*-test), respectively.

In the 2016–17 season, the dormancy states of 6-year-old DEX-treated GR21 and GR22 plants were also determined. Bud outgrowth was significantly retarded in DEX-treated GR21 and GR22 lines compared with the control plants (Fig 6). The DEX treatment did not affect the bud outgrowth rate or the phytohormone contents in terminal buds of WT plants (Fig 6). However, DEX-treated GR21 plants accumulated more ABA and less iP and tZ by 96 h after the treatment compared with the control plants (Fig 6). In particular, the iP content was significantly decreased by DEX at 24 h after the treatment. There were no significant differences between DEX-treated and control plants regarding the contents of the other phytohormones, IAA, JA, JA-Ile, and SA.

### Seasonal changes in the phytohormone contents of Japanese apricot dormant buds

Our above described analysis suggested that overexpression of *PmDAM6* represses bud break competency in 4- to 6-year-old transgenic apples and also increased ABA levels during dormancy and bud break stage and decreased cytokinin contents during bud break stage. To address whether *PmDAM6* also increased ABA levels and decreased cytokinins in Japanese apricot leaf and flower buds, we aimed to clarify the relationship between *PmDAM6* expression changes and phytohormone contents of Japanese apricot leaf and flower buds during endodormancy release toward bud break. Several previous studies reported *PmDAM6* was up-regulated during endodormancy and then down-regulated toward bud break in leaf buds and flower buds [4, 15, 18, 19].

**Fig 7:**
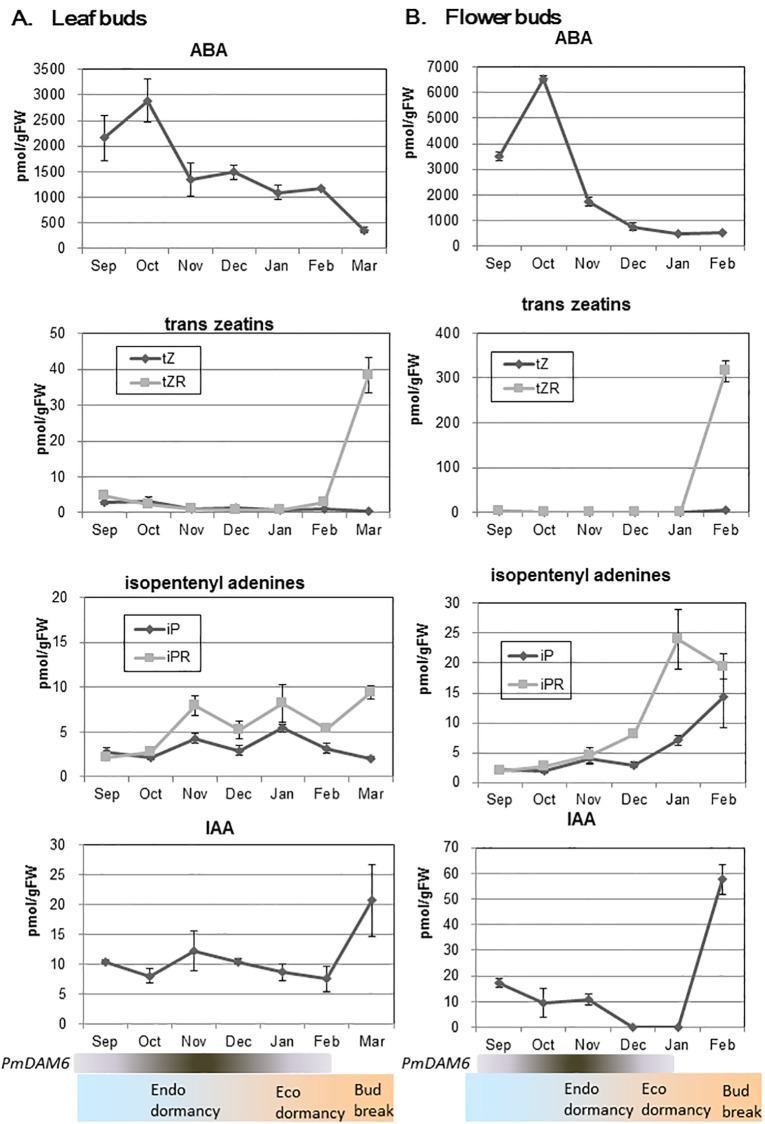
Seasonal changes in the phytohormone contents of dormant Japanese apricot ‘Nanko’ buds. The *PmDAM6* expression levels and dormancy status are briefly described at the bottom based on the previous studies. Values are presented as the mean of three replicates. Bars indicate standard deviations.

Previous studies revealed that Japanese apricot ‘Nanko’ flower and leaf buds gradually shift from endodormancy to ecodormancy in December and January and bud break occurs in February and end of March, respectively, under the climate conditions where samples were collected [15, 19]. In the current study, the ABA contents in buds steadily decreased after peaking in October (Fig 7). Decreases in the ABA content were synchronized with the shift from endodormancy to ecodormancy in both flower and leaf buds and also from ecodormancy to bud break in leaf buds. This suggested that the ABA concentrations in buds are associated with the depths of endodormancy and ecodormancy. The IAA contents decreased in buds as the season progressed, but increased just before bud break (Fig 7). The iP levels increased slightly during endodormancy release and increased further during ecodormancy release until bud break (Fig 7). The tZ contents did not undergo any major changes until February and January in leaf and flower buds, respectively, and then rapidly increased just before bud break (Fig 7). The results suggested that *PmDAM6* expression changes [15, 19] may be positively correlated with ABA contents in Japanese apricot leaf and flower buds. Additionally, increase in cytokinins was detected in bud break stage when *PmDAM6* expression was undetectable level in leaf and flower buds [15, 19]. Therefore, the obtained results appeared not contradictory to our hypothesis that *PmDAM6* may mediate increase and decrease in the abscisic acid and cytokinins contents, respectively, in dormant Japanese apricot buds.

## Discussion

To investigate the role of *PmDAM6* in dormancy regulation, we used apple, a relative of Japanese apricot, both belonging to Rosaceae, because there are easily transformable apple accessions [38]. However, the efficiency of the transformation of apple plants with the *p35S*:*PmDAM6* construct was very low, suggesting that *PmDAM6* may inhibit adventitious shoot regeneration in apple. The low regeneration efficiency after the overexpression of *SVP-like* or *DAM-like* genes in apple was also reported for *MdSVPa* and *MdDAMb* [35]. Nonetheless, two *PmDAM6*-overexpressing transgenic lines were obtained. We also generated transgenic apple lines that expressed a fusion between PmDAM6 and the hormone-binding domain of a glucocorticoid receptor (PmDAM6-GR). In these transgenic apple plants, PmDAM6-GR was activated by DEX treatments, as developed by Aoyama and Chua [37]. This system allowed us to evaluate the function of *PmDAM6* during dormancy release, while avoiding its effects on shoot growth prior to dormancy establishment.

### *PmDAM6* inhibited growth regardless of plant age and induced bud set in 4-year-old apple plants

The shoot growth of *35S*:*PmDAM6* apple plants was inhibited over two consecutive seasons under both LP and SP conditions. Differences in node numbers and internode lengths resulted in significantly different plant heights between WT and *35S*:*PmDAM6*. Thus, *PmDAM6* may inhibit shoot elongation by affecting both meristem activity and cell elongation. In response to a naturally-occurring cold conditions during the 2013–2014 season, 4-year-old *35S*:*PmDAM6* plants exhibited an earlier bud set compared with WT plants (Fig 2). The earlier bud set in 4-year-old *PmDAM6*-overexpressing apple plants was confirmed by the bud-set timing of the DEX-treated GR lines (S3 Table). Thus, dormancy induction might be affected by the overexpression of *PmDAM6* in apple. In peach, *DAM* genes are thought to be responsible for terminal bud set [14]. In fact, peach *DAM6* is up-regulated when terminal bud set is observed in the field [44]. In the perennial herbaceous species leafy spurge (*Euphorbia esula*), the *DAM* homologs *DAM1* and *DAM2* are associated with dormancy induction [45]. Indeed, early bud set has been observed in *35S*:*PmDAM6* poplar [15]. In contrast, transgenic apple plants overexpressing *MdDAMb* do not exhibit early bud set [35], although the phenotypes of *35S*:*MdDAMb* plants were observed only during the first 2 years. In the current study, the *35S*:*PmDAM6* apple plants did not exhibit earlier bud set during the first 2 years. Dormancy induction and establishment may be controlled by a complex genetic mechanism that is affected by environmental conditions [46]. Although this study demonstrated that *PmDAM6* promotes dormancy entry in apple, future studies should aim to clarify whether *PmDAM6* also influences dormancy establishment as discussed below.

### *PmDAM6* inhibited lateral bud break, but accelerated terminal bud break in 3-year-old apple plants

When 3-year-old *35S*:*PmDAM6* apple plants were grown under semi-field conditions, the lateral bud break was inhibited (Fig 3), while the terminal bud break was advanced relative to that of the WT plants. Moreover, the DEX-treatment of GR plants also accelerated the terminal bud break and inhibited lateral bud break (Table 1). This phenotype was observed only in 3-year-old plants consisting of a single 1-year-old shoot. In contrast, older plants bearing multiple 1-year-old shoots exhibited delayed terminal bud break (Table 2). The mechanisms underlying the *PmDAM6*-mediated inhibition of the lateral bud break and acceleration of the terminal bud break in 3-year-old plants, remain unclear. One possibility is that *PmDAM6*-induced growth inhibition may be antagonized with a strong sink activity at the terminal buds that take up water and nutrients. Consequently, only the lateral buds exhibit inhibited bud break in *PmDAM6* overexpressing lines. Alternatively, *PmDAM6* may directly inhibit only the lateral buds and not the terminal buds. In fact, *SVP-like* in hybrid poplar, *SVL* induces *TCP18/BRC1* which suppress lateral bud break [47]. Another possible explanation is that *PmDAM6* may enhance apical part dominance effects during the bud break stage in 3-year-old plants. Faust et al. [48] conducted decapitation experiments using apple dormant shoots. They concluded that apical dominance effects occur during the later phase of apple dormancy, and that a prolonged chilling exposure releases both apical dominance and endodormancy. Recently, Yao and Finlayson proved that ABA restricts the outgrowth of lower buds and promotes correlated inhibition [49]. These findings raise the possibility that ABA is one of the first upstream factors regulating the apical dominance responses to the red:far red ratio [50]. As discussed below, the overexpression of *PmDAM6* increased the days to bud break in forcing condition, implying that *PmDAM6* enhanced the dormancy depth of terminal buds during the adult phase. Additionally, *PmDAM6* increased the ABA contents in apple terminal buds. Accordingly, we can hypothesize that *PmDAM6* inhibits bud break in 3-year-old plants only in the lower lateral buds, possibly by increasing ABA contents. This hypothesis will need to be tested in future studies. Nevertheless, it remains unclear why this phenotype was observed only in 3-year-old plants and not in older plants.

### *PmDAM6* represses bud break competency and delays bud outgrowth via the accumulation of ABA and decrease in cytokinins contents in terminal buds of 4- to 6-year-old apple plants

**Fig 8:**
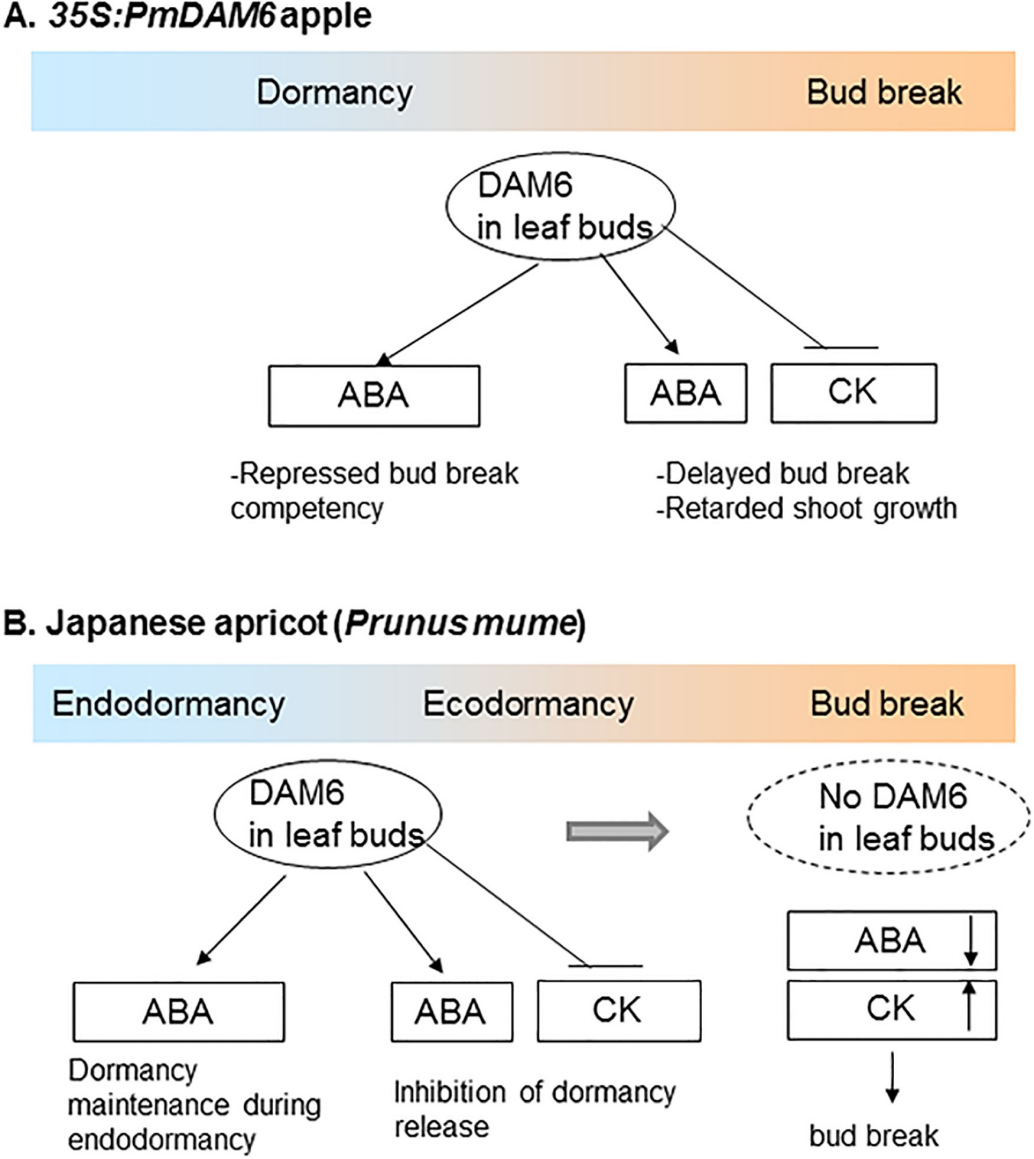
Schematic diagram of the proposed model of *PmDAM6* functions during dormancy and bud outgrowth in *PmDAM6*-overexpressing transgenic apple plants and in Japanese apricot. (A) Proposed model of *PmDAM6* functions in *35S*:*PmDAM6* apple plants. *PmDAM6* induces the accumulation of ABA and inhibits the accumulation of cytokinins (CK) in dormant terminal buds, which represses bud break competency and inhibited bud outgrowth. (B) Model of *PmDAM6*-mediated dormancy regulation in Japanese apricot based on data obtained from transgenic apple plants (this study), seasonal changes in *PmDAM6* expression levels [15, 19] and seasonal changes in phytohormone contents (this study) in Japanese apricot leaf buds. The expression of *PmDAM6* is up-regulated during endodormancy and down-regulated during the endo- and ecodormancy release stages. Additionally, *PmDAM6* expression is undetectable during bud break [15, 19]. Down-regulated *PmDAM6* expression coincides with a decrease in ABA during the endo- and ecodormancy release stages and with an increase in CK levels during the ecodormancy release and bud break stages. We hypothesize that *PmDAM6* maintains endodormancy by repressing bud break competency and increasing ABA levels and inhibits dormancy release by increasing ABA and decreasing CKs contents in dormant Japanese apricot buds.

The overexpression of *PmDAM6* delayed lateral and terminal bud break in apples after 4 years under semi-natural conditions (Table 2, Fig 4). Additionally, the dormancy depth was increased owing to the overexpression of *PmDAM6* in apple (Table 2). This is consistent with the results of a previous study on transgenic kiwifruit overexpressing *SVP2*, in which it prevented premature bud break [51]. If *PmDAM6* is an important regulator for dormancy release, then it may induce major physiological changes in dormant buds. Thus, we investigated the phytohormone contents of the terminal buds from transgenic plants. Among the phytohormones, ABA is important for winter bud dormancy in woody perennials and the axillary bud dormancy of herbaceous species [1, 49, 50, 52, 53]. Our data suggest that *PmDAM6* enhanced dormancy and delayed bud break, while simultaneously inducing the accumulation of ABA in the terminal buds of transgenic apple plants (Fig 5). Moreover, the ABA concentration was higher during the endodormant period and decreased during endodormancy release in Japanese apricot buds (Fig 7). We previously reported that *PmDAM6* expression is down-regulated during endodormancy release toward bud break [15, 19]. These findings suggest the seasonal down-regulation of *PmDAM6* expression coincides with a decrease in the ABA contents during endodormancy release and bud break (Fig 7) in Japanese apricot leaf buds. Therefore, we hypothesize that *PmDAM6* contributes to enhance the dormancy of Japanese apricot buds by mediating an increase in ABA contents (Fig 8). Tuan et al. [54] reported that pear *PpDAM1* up-regulates the expression of the gene encoding 9-cis-epoxycarotenoid dioxygenase (NCED) by binding to the CArG motif in the promoter. Additionally, high levels of hybrid poplar *SVP-like*, *SVL* expression induces *NCED3* [47]. Thus, *PmDAM6* may contribute to the accumulation of ABA in terminal buds by promoting ABA biosynthesis. Although Wu et al. [51] did not find major differences in the ABA concentrations of *35S*:*SVP2* and control kiwifruit plants, their RNA-Sequencing analysis suggests that ABA and dehydration response pathways are modulated by the overexpression of *SVP2*. Thus, some *SVP/DAM*s genes may have conserved functions regarding the inhibition of bud break in poplar and several temperate fruit tree species during dormancy release and the bud break stage. These functions may involve modulating ABA accumulation and/or signaling.

During the bud break stage, the overexpression of *PmDAM6* increased ABA and decreased cytokinins levels in the terminal buds of apple plants (Fig 5). To the best of our knowledge, this is the first report of a possible interaction between *DAM* genes and cytokinins in woody plants. Cytokinins generally facilitate cell division and elongation, as well as bud outgrowth [55]. In fact, auxin and cytokinins levels increase in buds during the late stages of ecodormancy release toward bud break in Japanese apricot, suggesting that they influence bud outgrowth rather than dormancy. Additionally, in *Rosa hybrida*, ABA and cytokinins may function antagonistically during the regulation of axillary bud break in response to light intensity [56]. We observed that ABA contents continue to decrease not only from endodormancy to ecodormancy but also from ecodormancy to bud break in Japanese apricot leaf buds (Fig 7). Recently, Kitamura et al. [8] suggested that *PmDAM6* transcripts levels at ecodormant stage are associated with the days to bud break in forcing condition in one F_1_ population of Japanese apricot. Therefore, *PmDAM6* may affect bud break competency during the endodormancy release stage as well as the bud break stage. Our results presented herein suggest that *PmDAM6* may retard bud outgrowth by mediating increasing ABA and decreasing cytokinins contents in buds during the bud break stage (Fig 8).

### *PmDAM6* may be a growth inhibitor and act as a lateral bud break repressor in Japanese apricot buds

Our study demonstrated that *PmDAM6* inhibits plant regeneration and growth, and promotes bud set, as well as represses bud break competency in transgenic apple plants. Additionally, *PmDAM6* inhibits lateral bud break, as well as terminal bud break with the exception of those in young transgenic apple plants. Moreover, a working hypothesis regarding how *PmDAM6* affects dormancy release and bud break in Japanese apricot has been developed based on the results of this study (Fig 8). Specifically, *PmDAM6* might inhibit dormancy release and maintain dormancy by inducing the accumulation of ABA in dormant buds. Additionally, it may delay bud outgrowth by affecting the balance of endogenous ABA and cytokinins, which function antagonistically in the regulation of bud break and bud outgrowth. Our data provide a foundation for future analyses of *PmDAM6* functions affecting the complex cross-talk between ABA and cytokinins that regulates dormancy and bud break. The putative role of *PmDAM6* in repressing lateral bud break competency during dormancy revealed in this study appears to be consistent with the finding that *PmDAM6* expression is up-regulated and down-regulated during “endodormancy” induction and release, respectively [15]. According to Sasaki et al. [15], “endodormancy” induction and release were estimated based on the decrease and increase in bud break frequency and/or the decrease and increase in the number of days to bud break under forcing conditions, respectively. Because all *PmDAM*s were expressed when bud break competency is repressed in lateral leaf buds [15], we hypothesize that all *PmDAM*s may share the conserved bud break repressor function as *PmDAM6* does. However, *SVP* clade where *PmDAM*s belong to is supposed to be under positive selection [57], exact role of other *PmDAM*s should be elucidated by future genetic studies. Although the present study suggested the possible involvement of *PmDAM6* in dormancy entry, it still remains unclear whether *PmDAM6* also affects dormancy establishment. Considine and Considine [5] recently proposed changes to dormancy terminology, and suggested that bud dormancy can be further divided into either dormant (D) or quiescent (Q) states depending on the chromatin state in dormant meristem [i.e., heterochromatin (D state) or euchromatin (Q state)]. To verify the possible involvement of *PmDAM6* for dormancy establishment, we may have to first determine whether D state cells exist in *Prunus* (and other fruit tree species in Rosaceae) dormant buds or not, then if any, the critical time points when the D state begins and ends in the leaf bud meristem.

In poplar, ABA mediates short-photoperiod induction of *SVP-like*, *SVL* expression during bud dormancy establishment [58]. In Rosaceae, dormancy of *Prunus* species is induced by the interaction of short photoperiod and low temperature [59]. In contrast, apple is typically not sensitive to short photoperiod for dormancy induction [60]. However, Wisniewski et al. [61] reported that peach *CBF*-overexpressing apple is sensitive to short photoperiod and induced bud set, which suggests that short photoperiod-induced dormancy pathway may be, at least in part, conserved in rosaceous fruit tree species. Additionally, our previous investigation indicated there is a close relationship between *PmDAM6* transcripts levels and ABA contents in flower buds of two Japanese apricot cultivars with contrasting chilling requirement for dormancy release [19, 62]. These results collectively suggested that Japanese apricot dormancy may be at least partially induced by short photoperiod and ABA-dependent, which appeared to be similar to the *SVL*-mediated poplar dormancy. If the *PmDAM6*-mediated dormancy release pathway is somehow similar to *SVL*-mediated dormancy regulation in poplar [47, 58, 63], ABA biosynthesis and/or signaling may be a putative upstream key factor for *PmDAM6*-mediated regulation of dormancy and bud break in Japanese apricot.

## Supporting information

S1 Fig Confirmation of apple transformants overexpressing *PmDAM6*. (PPTX)

S2 Fig Three-year-old plants grown under short photoperiod (8-h light/16-h dark) conditions. The current year’s shoot growth was inhibited in the 35S:PmDAM6 lines compared with WT.
(TIF)

S1 Table Primer sequences used for RT-qPCR analyses of transgenic apple plants. (DOCX)

S2 Table Node numbers and internode lengths of 3-year-old WT and *35S*:*PmDAM6* apple plants. (XLSX)

S3 Table Inhibited shoot growth and early bud set observed in 4-year-old *35S*:*PmDAM6-GR* apple plants treated with dexamethason (DEX). (XLSX)

S4 Table Terminal bud break date of 3- to 6-year-old plants and the number of open lateral buds in 3-year-old WT and *35S*:*PmDAM6* apple plants. (XLSX)

S5 Table Terminal bud break date and the number of open lateral buds in 4-year-old *35S*:*PmDAM6-GR* plants. (XLSX)

S6 Table Repressed bud break competency of 5- and 6-year-old WT and *35S*:*PmDAM6* apple plants. (XLSX)

S7 Table Phytohormone contents in the terminal buds of 4-year-old plants collected on 20 February 2015. (XLSX)

S8 Table Phytohormone contents in the terminal buds of 6-year-old plants collected on 25 January, 23 February, and 8 and 31 March 2017. (XLSX)

S9 Table Bud outgrowth rate of the branches of 6-year-old *35S*:*PmDAM6-GR* plants. (XLSX)

S10 Table Phytohormone contents in the terminal buds of DEX-treated or control WT and GR21 plants at 0, 24, and 96 h after treatment. (XLSX)

S11 Table Phytohormone contents of Japanese apricot ‘Nanko’ leaf and flower buds. (XLSX)
