# Supplementary material for: Overexpression of Prunus DAM6 inhibits growth, represses bud break competency of dormant buds and delays bud outgrowth in apple plants
Source: PLoS One. 2019 Apr 9;14(4):e0214788. doi: 10.1371/journal.pone.0214788 (PMC6456227; doi:10.1371/journal.pone.0214788)
Supplement: S1 Fig — (PPTX) [file pone.0214788.s001.pptx]

## Slide 1
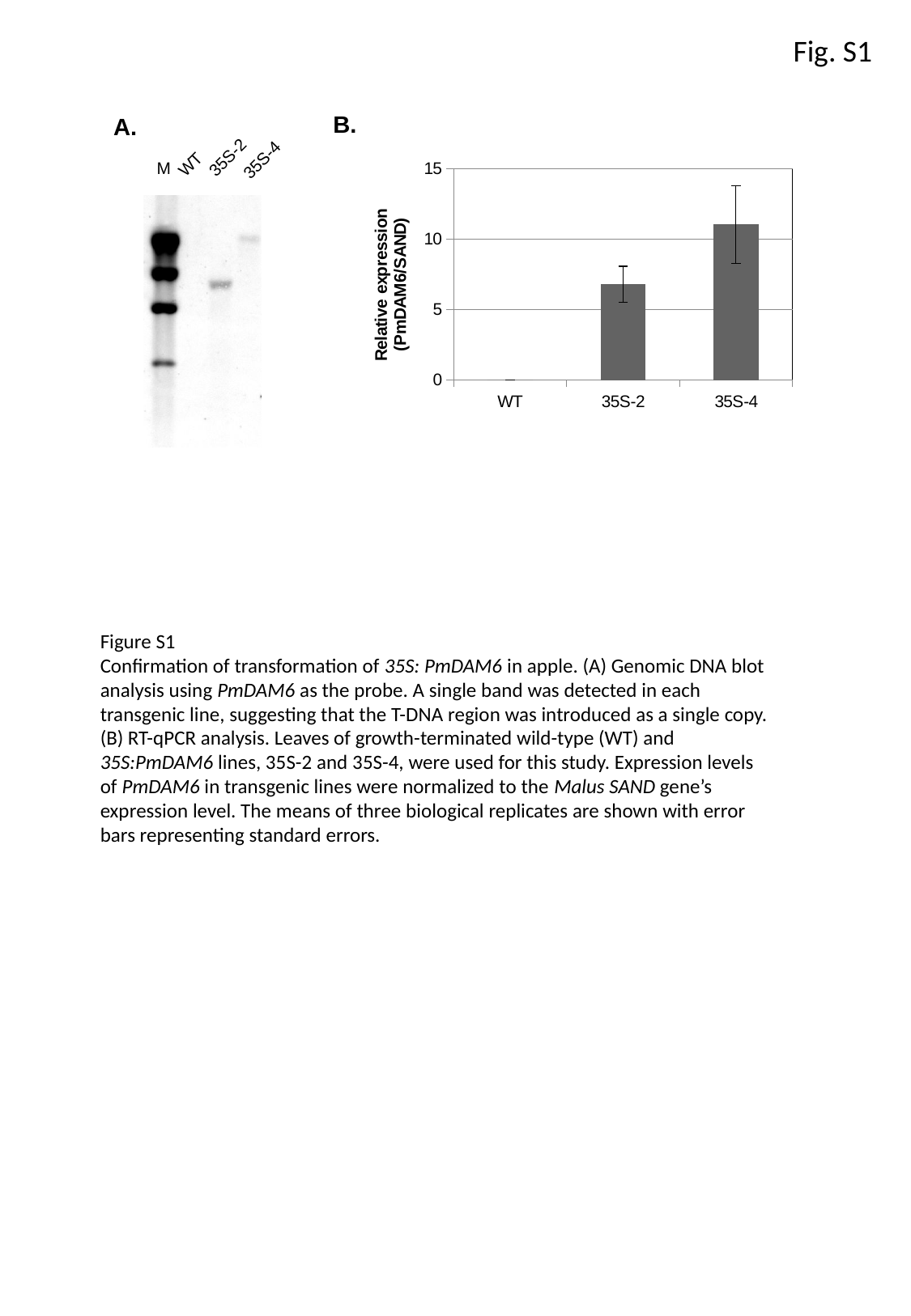

Fig. S1
B.
A.
35S-2
WT
35S-4
 M
### Chart
| Category | |
|---|---|
| WT | 0.0 |
| 35S-2 | 6.793144454037633 |
| 35S-4 | 11.035021413600267 |
Figure S1
Confirmation of transformation of 35S: PmDAM6 in apple. (A) Genomic DNA blot analysis using PmDAM6 as the probe. A single band was detected in each transgenic line, suggesting that the T-DNA region was introduced as a single copy. (B) RT-qPCR analysis. Leaves of growth-terminated wild-type (WT) and 35S:PmDAM6 lines, 35S-2 and 35S-4, were used for this study. Expression levels of PmDAM6 in transgenic lines were normalized to the Malus SAND gene’s expression level. The means of three biological replicates are shown with error bars representing standard errors.
