# Supplementary figures and images for: Overexpression of Prunus DAM6 inhibits growth, represses bud break competency of dormant buds and delays bud outgrowth in apple plants
Source: PLoS One. 2019 Apr 9;14(4):e0214788. doi: 10.1371/journal.pone.0214788 (PMC6456227; doi:10.1371/journal.pone.0214788)

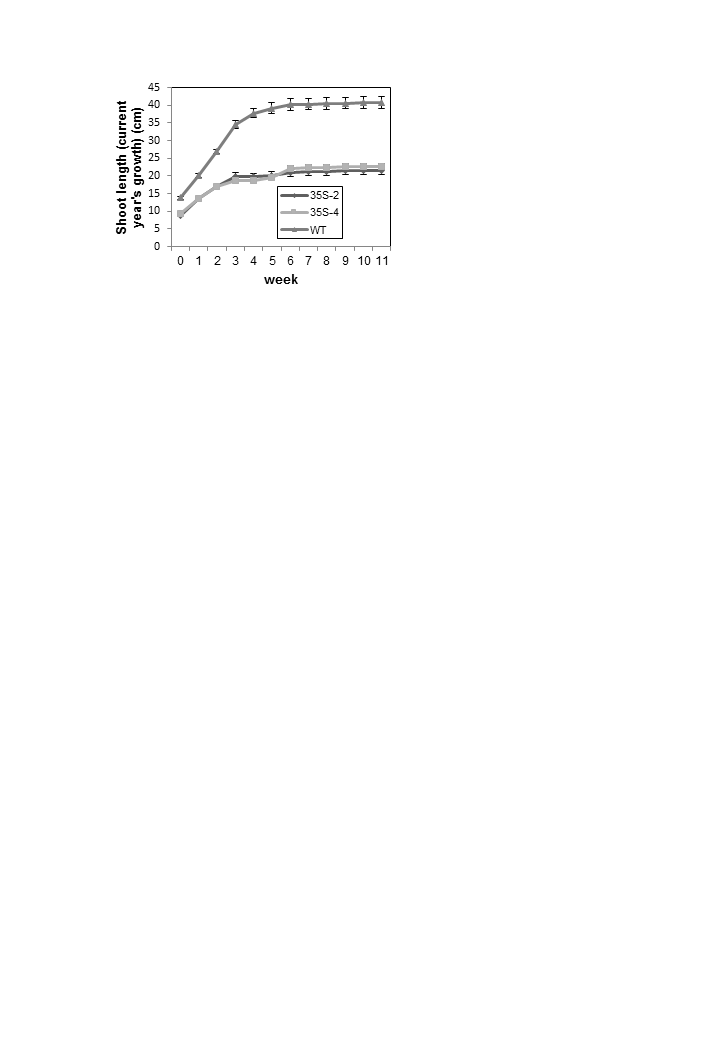

Supplement: S2 Fig — The current year’s shoot growth was inhibited in the 35S:PmDAM6 lines compared with WT. (TIF) [file pone.0214788.s002.tif]
