# Supplementary material for: Overexpression of Prunus DAM6 inhibits growth, represses bud break competency of dormant buds and delays bud outgrowth in apple plants
Source: PLoS One. 2019 Apr 9;14(4):e0214788. doi: 10.1371/journal.pone.0214788 (PMC6456227; doi:10.1371/journal.pone.0214788)
Supplement: S1 Table — (DOCX) [file pone.0214788.s003.docx]

Supplementary Material

Table S1. Primer sequences used for this study

Target gene name Primer/probe name Sequence Reference

*PmDAM6* PmDAM6_F2 5’-ACCAGCTGCAACAGTGCTTT-3’ Sasaki et al. (2011)

PmDAM6_R2 5’-ACTAGGGAAGCCCCAGTTTG-3’

PmDAM6-T (probe) 5’-(FAM)-TGAAGATGACTGCTC

CGATGTCACTTTATC-(TAMRA)-3’

*MdSAND* MalusSAND_qp_F 5’-GTTGCCGATTCTGGTGTTCT-3’ this study

MalusSAND_qp_R2 5’-TGAAGAGAGGCCTTTTGAGCA-3’
